# Synthetic CTs for MRI-only brain RT treatment: integration of immobilization systems

**DOI:** 10.1007/s00066-023-02090-w

**Published:** 2023-06-07

**Authors:** Siti Masitho, Johanna Grigo, Tobias Brandt, Ulrike Lambrecht, Juliane Szkitsak, Alexander Weiss, Rainer Fietkau, Florian Putz, Christoph Bert

**Affiliations:** 1 Department of Radiation Oncology, Strahlenklinik, Universitätsklinikum Erlangen, Friedrich-Alexander-Universität Erlangen-Nürnberg (FAU), Universitätsstraße 27, 91054 Erlangen, Germany; 2 Comprehensive Cancer Center Erlangen-EMN (CCC ER-EMN), Erlangen, Germany

**Keywords:** Stereotactic radiosurgery, Artificial intelligence, Treatment planning, Image-guided radiotherapy, Radiotherapy setup

## Abstract

**Purpose:**

Auxiliary devices such as immobilization systems should be considered in synthetic CT (sCT)-based treatment planning (TP) for MRI-only brain radiotherapy (RT). A method for auxiliary device definition in the sCT is introduced, and its dosimetric impact on the sCT-based TP is addressed.

**Methods:**

T1-VIBE DIXON was acquired in an RT setup. Ten datasets were retrospectively used for sCT generation. Silicone markers were used to determine the auxiliary devices’ relative position. An auxiliary structure template (AST) was created in the TP system and placed manually on the MRI. Various RT mask characteristics were simulated in the sCT and investigated by recalculating the CT-based clinical plan on the sCT. The influence of auxiliary devices was investigated by creating static fields aimed at artificial planning target volumes (PTVs) in the CT and recalculated in the sCT. The dose covering 50% of the PTV (D_50_) deviation percentage between CT-based/recalculated plan (∆D_50_[%]) was evaluated.

**Results:**

Defining an optimal RT mask yielded a ∆D_50_[%] of 0.2 ± 1.03% for the PTV and between −1.6 ± 3.4% and 1.1 ± 2.0% for OARs. Evaluating each static field, the largest ∆D_50_[%] was delivered by AST positioning inaccuracy (max: 3.5 ± 2.4%), followed by the RT table (max: 3.6 ± 1.2%) and the RT mask (max: 3.0 ± 0.8% [anterior], 1.6 ± 0.4% [rest]). No correlation between ∆D_50_[%] and beam depth was found for the sum of opposing beams, except for (45° + 315°).

**Conclusion:**

This study evaluated the integration of auxiliary devices and their dosimetric influence on sCT-based TP. The AST can be easily integrated into the sCT-based TP. Further, we found that the dosimetric impact was within an acceptable range for an MRI-only workflow.

## Introduction

For brain radiotherapy (RT) patients are commonly immobilized using an RT mask system, typically consisting of an RT thermoplastic mask, an RT mask holder, and an RT flat tabletop. This setup is applied during CT scans and during treatment at the linear accelerator [1, 2]. In CT-based RT planning, these auxiliary devices are considered in the dose calculation, as radiation can penetrate through the devices from certain angles, and couch tops and immobilization devices have been reported to have dosimetric effects [3]. Some of these devices can be defined in the CT scan and their Hounsfield units (HU) translated directly as electron density in the treatment planning system (TPS) if they are identical to the ones used at the treatment machine. However, the material and shape of the tabletop and mask holder at the CT sometimes differ from the devices used at the treatment machine. In this case, these devices’ structures can, for example, be added retrospectively as structure templates during treatment planning or automatically contoured [3, 4].

In a typical CT-based workflow, it has been reported that magnetic resonance imaging (MRI) acquisition setup may lead to MRI–CT registration inaccuracy [5, 6]; nonetheless, acquiring MRI in the RT setup can reduce this inaccuracy [1]. This can set a challenge for the implementation of synthetic CT (sCT) for an MRI-only workflow. Also there, an RT setup has to be used for MRI scans [1, 5, 7, 8], especially since the sCT then defines the treatment position. This typically requires an MR-compatible RT flat tabletop, RT thermoplastic mask, a mask holder, and a dedicated coil setup [9–13]. In our institution, the RT mask holder and RT tabletop available at the CT and the treatment machine cannot be used for MRI due to their MR incompatibility. Hence, a wooden mask holder was built for this purpose [1, 2]. The accuracy of this setup should be regularly assured so that no large difference between the patient’s position in MRI and treatment can occur [1, 8].

While a previous in silico study assessing the dosimetric differences between sCT- and CT-based plans has been conducted and an equivalent dose distribution to a CT-based plan was reported [8], the limitation of this study was that the auxiliary devices were not considered in the dose calculation. The challenge for implementation of an MRI-only workflow is that these devices are not visible in the MR images. Defining these devices in the sCT is crucial for MRI-based clinical treatment planning. Other studies evaluating brain or head and neck sCTs acquired MRI in the RT position but did not consider most auxiliary devices in the dose evaluation [10, 12–15]. Meanwhile, in the following studies [16–18], the MRI was not acquired in the RT position. Since the auxiliary devices may have an additional influence on the dose calculation, the influence is yet to be addressed before full clinical implementation of the sCT-based treatment planning.

Treatment planning MRI can include T2-FLAIR, T1-SPACE [9], and also T1-VIBE DIXON for generation of the sCT [19]. Additional sequences to visualize the auxiliary devices in MRI such as ultrashort echo time (UTE) can be acquired [10], but this may prolong the acquisition time. Due to the required long high-resolution sequences for brain RT [5, 9], acquisition time per patient should be kept to a minimum. Most importantly, in this study, the shape and material of the mask holder and tabletop are different from the ones used for treatment, thus acquiring extra sequences to directly define these devices’ structures on the MRI is redundant, except for determining their position relative to the patient, as done in [10]. In our case, the indirect approach of modeling the auxiliary devices retrospectively during treatment planning based on the relative position can be useful, for example by using embedded MRI-visible markers.

For clinical implementation of sCTs in an MRI-only workflow, acceptable dose differences between sCT and the CT-based plans need to be assured. In previous studies, the dose calculations were done without considering the auxiliary devices. Hence, in this work, a technical solution for full implementation of sCTs in a clinical MRI-only workflow was introduced, such as definition of the immobilization system and RT table in the sCT for treatment planning. Further, the dosimetric influence of the auxiliary devices was evaluated.

## Materials and methods

### RT setup preparation

**Fig. 1:**
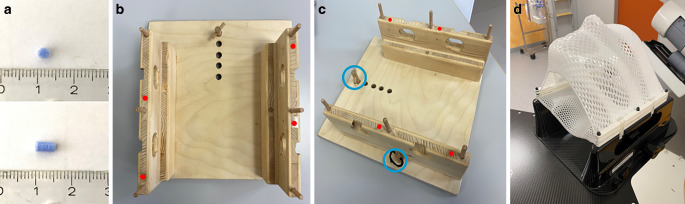
Radiotherapy (RT) setup preparation. **a** A cylindrical silicon marker with a height of 5 mm and a diameter of 3 mm. **b** In-house built mask holder made out of wooden material. **c** Fixations (*blue circle*) for positioning on the RT table can be found on top (1 piece) and on the sides (2 pieces). *Red dots* indicate the position of silicone markers that were embedded in the mask holder. **d** RT mask fixed on the treatment table

RT setup was used for the MRI, CT, and treatment. In the MRI this includes the RT mask (basic cranial mask; Brainlab, Munich, Germany) and a wooden mask holder. The RT mask consist of two parts (upper and bottom part). To improve patient immobilization, an extra thermoplastic material can be additionally molded between the nose and the RT mask (Fig. 1d).

MRI-visible markers were created out of soft silicone (DUOSIL H; SHERA, Werkstoff Technologie, Lemförde, Germany) and formed using a 3D-printed mold. The markers have a cylindrical form (height = 5 mm, diameter = 3 mm; Fig. 1a). In total, four markers were embedded inside the mask holder as depicted in Fig. 1b, c. Fixations for optimal positioning of the mask system on the RT tabletop can be found on top (1 piece) and on the sides (2 pieces).

### Auxiliary structure template definition

**Fig. 2:**
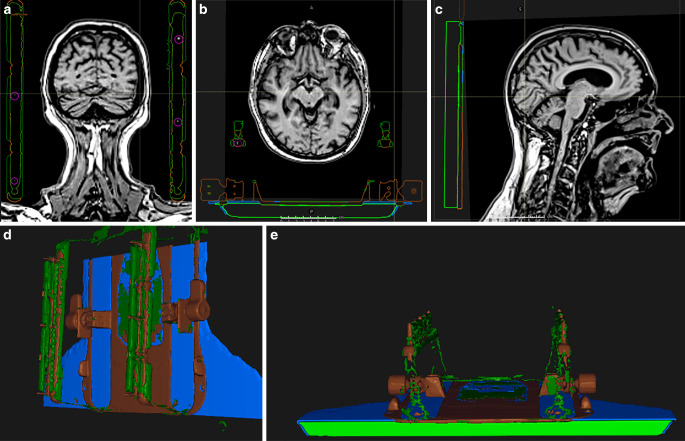
Auxiliary structure template (AST). **a**, **b**, **c** An example of the AST position on coronal, axial, and sagittal views of a typical T1-VIBE DIXON, **d**, **e** 3D representation of the AST. *Magenta* markers, *green* mask holder (low density), *brown* mask holder (high density), *light green *RT tabletop (low density), *blue* RT tabletop (high density). Signal from the silicone markers can be seen inside the markers’ region of interest

To determine the relative position between markers and mask, initially, CT scans (1 mm isotropic resolution) of both the MRI mask holder and the RT mask holder were acquired and rigidly registered based on the RT mask structure. The auxiliary structure template (AST; Fig. 2) was then defined in the treatment planning system (RayStation 10B; RaySearch, Stockholm, Sweden) as a voxel-based volume. It consists of:

1. four circular regions-of-interest (ROIs) with a diameter of 5 mm centered at the position of the MR-visible markers;
2. mask holder structure made of two materials with densities of 1.2 g/cm^3^ and 1 g/cm^3^;
3. RT table made of two different materials with densities of 0.11 g/cm^3^ and 1.7 g/cm^3^.

The relative mass densities of the AST were based on the HU values determined in the acquired CT, which were then converted to each respective mass density based on the HU look-up table (HU LUT).

### Patient data and sCT generation

For this evaluation, a retrospective patient study was conducted, which is an extension of the first study (for details see [8]). Included were data from 20 consecutive patients receiving brain RT treatment in the period between June and September 2022 using the Brainlab RT mask system, from which 10 datasets of patients with prosthetics, e.g., eye prosthetics, were excluded (in total 10 patients, for details see Table 1). The T1‑w VIBE DIXON (1.5 mm × 1.5 mm × 1.5 mm) sequence was acquired at the 1.5T MRI (MAGNETOM Sola, Siemens Healthineers, Erlangen, Germany) as part of the clinical protocol. The field of view of the T1-VIBE DIXON was sufficiently large to ensure the markers’ inclusion in the image. Syngo.via VB60 RT pro edition (Siemens Healthineers, Erlangen, Germany) was used to generate sCTs using a deep neural network-based algorithm [8, 19]. From the 10 patients’ data, a subset of four patients was randomly chosen for the evaluation of RT mask material (P01, P02, P03, P04), and four additional patients were chosen for the evaluation of AST position (P03, P04, P05, P09). All procedures performed were in accordance with the ethical standards of the institutional research committee and with the 1964 Helsinki declaration and its later amendments. Patient consent was not required for this retrospective study per institutional policy and in accordance with local legislation (BayKrG Art. 27 (4)).

**Table 1:** Patient data

Patient ID	Gender	Age (years)	Tumor entity	PTV size (cc)	Fractionation	Irradiation technique
P01	F	68	Frontal right	16.02	12 × 500 cGy	VMAT
P02	M	61	Frontal right	84.48	28 × 200 cGy	VMAT
P03	F	73	Multiple metastases	2.33	12 × 500 cGy	VMAT
P04	M	60	Cerebellum right	14.59	28 × 200 cGy	VMAT
P05	F	81	Multiple metastases	5.75	12 × 500 cGy	VMAT
P06	F	59	Whole brain	1675.00	12 × 300 cGy	VMAT
P07	F	60	Acoustic neuroma left	0.89	1 × 1625 cGy	Static arc
P08	F	59	Whole brain	1834.50	10 × 300 cGy	VMAT
P09	M	70	Parieto-occipital right	0.48	1 × 2500 cGy	Static arc
P10	M	65	Cerebellum left	0.34	1 × 2500 cGy	Static arc
*Median*	*–*	*63*	*–*	*10.17*	*–*	*–*

*PTV* Planning Target Volume, *VMAT* Volumetric Modulated Arc Therapy

The AST was loaded onto the T1‑w VIBE DIXON (opposed-phase contrast) of each patient. Afterwards, the AST was translated and rotated until the MR-visible markers were inside each marker ROI. With the appropriate windowing of level/width = 100/200 in the TPS, the markers were visible.

### Data analysis

After molding, the RT mask material can have varying thicknesses throughout. Further, the RT mask’s density uncertainty should also be considered. In the first part of the evaluation, the influence of mask material was evaluated by assigning an RT mask structure with different mask thicknesses (*d* = 2.5 mm, 3 mm) and densities (ρ = 1.1 g/cm^3^, 1.15 g/cm^3^, 1.2 g/cm^3^) on four sCTs (P01, P02, P03, P04). First, the “external” structure, which defines the outer contour of the head, was expanded by a specified thickness *d*. Using ROI algebra “external + *d*” − “external,” a surrounding mask was defined. Second, the mask was assigned with a density ρ*. *The mask as well as the table and the mask holder structures were defined as ROIs and considered in the dose calculation.

Based on the results of the first evaluation, an RT mask was defined in all sCTs with the chosen optimal mask material. Clinical plans were recalculated using the corresponding sCT image dataset. D_50_ was evaluated to minimize inaccuracies caused by inhomogeneities. The mean deviation percentage (∆D_50_[%]) and mean deviation ∆D_50_ of the target volumes and various organs at risk (OARs) were calculated.

**Fig. 3:**
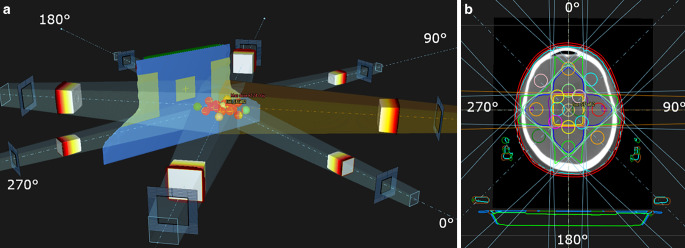
Static field test. Sixteen artificial planning target volumes (PTVs) with diameter 2 cm can be seen. **a** Static fields from eight different angles (0°, 45°, 90°, 135°,180°, 225°, 270°, 315°) aimed at the artificial PTV volumes; **b** the axial view of the beams, PTVs, dose distribution, and the auxiliary structure template in the sCT. The *blue/light green* structures represent the RT table, the *green/brown* structures the mask holder, and the *red structure *the RT mask

In the second part of the evaluation, the influence of the RT mask, mask holder, and RT table was evaluated by creating rectangular static fields from eight different beam angles: ([5 cm × 5 cm]: 0°, 90°, 180°, 270°; [10 cm × 10 cm]: 45°, 135°, 225°, 315°; each 1000 MU) in the CT of all patients, which were then recalculated in the sCT. Sixteen artificial PTVs (diameter = 2 cm) were defined on all patients in a pattern as seen in Fig. 3. The PTVs on the corresponding central axis of the beams were evaluated: for each opposing beam direction, four PTV spheres were distributed regularly through the brain. The influence of the RT mask was evaluated using the static field with beam angles of 0°, 90°, and 270°; the influence of the RT table using a static field with a beam angle of 180°; and the influence of the mask holder using static fields of beam angle 45°, 135°, 225°, and 315°.

The mean and standard deviation of the distance between the centroid of the PTV and the point of beam entrance (*D*_*PTV*_) were calculated, considering the variety of the PTV locations for each patient due to different head sizes. ∆D_50_[%] was evaluated for each beam angle and the sum with its opposite beam. To further evaluate the influence of AST position, the AST of four patients (P03, P04, P05, P09) was additionally repositioned based on the visible auxiliary devices on their corresponding CT image and the results were compared to the MR-visible markers-based positioning results.

## Results

### RT mask material

**Fig. 4:**
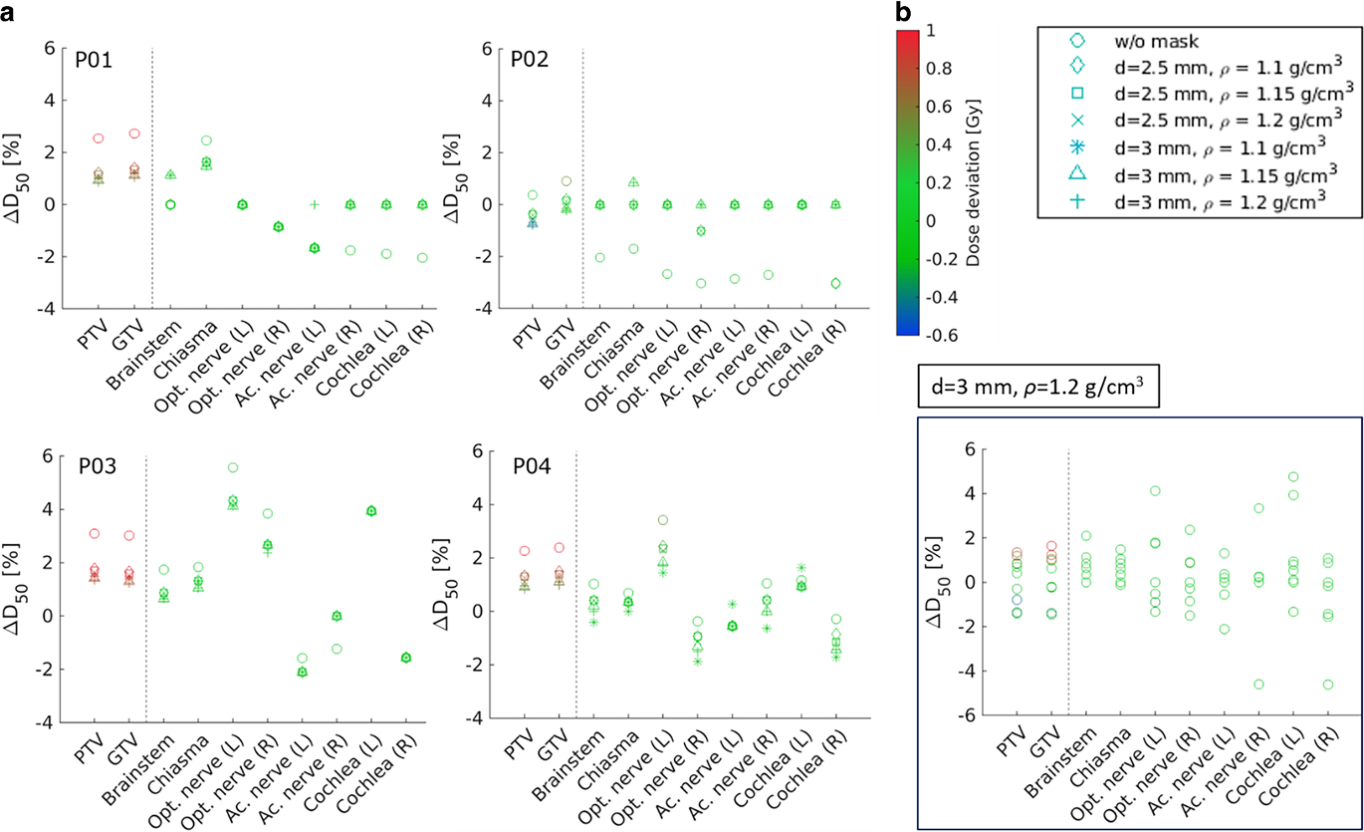
Dosimetric variation caused by the RT mask structure defined in the sCT-based dose calculation. The percentage deviation of D_50_ (∆D_50_[%]) between the CT-based clinical plan and recalculated clinical plan using the sCT is presented. The *colormap* shows the absolute dose deviation in Gy. **a** Variation of mask thickness (d) and density (ρ) on four exemplary patients. **b** Differences in all 10 patients using chosen mask specification (d = 3 mm and ρ = 1.2 g/cm^3^). *PTV* Planning Target Volume, *GTV* Gross Tumor Volume

The mean difference in D_50_ of the PTV between sCT-based and CT-based plans was largest when no mask was defined in the sCT (∆D_50_[%] = 2.1%, ∆D_50_ = 1.2 Gy) and decreased with increasing mask thickness and density (Fig. 4a). The smallest ∆D_50_[%] was found for *d* = 3 mm, ρ = 1.2 g/cm^3^ (∆D_50_[%] = 0.6%, ∆D_50_ = 0.3 Gy). Increasing the mask thickness by 0.5 mm reduced the ∆D_50_[%] by 0.3%, while increasing the assigned density by 0.05 g/cm^3^ reduced the ∆D_50_[%] by 0.06% for *d* = 3 mm, and by 0.02% for *d* = 2.5 mm. For OARs, the ∆D_50_[%] ranged between −1.7% and 1.6% for no mask, and the smallest ∆D_50_[%] range was found for *d* = 3 mm, ρ = 1.2 g/cm^3^ (−0.7% and 1.4%).

Since using a mask with *d* = 3 mm, ρ = 1.2 g/cm^3^ delivered the smallest ∆D_50_, this mask specification was defined for the analysis in all 10 patients. Evaluating the clinical plan, the mean ∆D_50_[%] was 0.2 ± 1.03% (0.2 ± 0.4 Gy) for PTV, 0.3 ± 1.1% (0.3 ± 0.5 Gy) for GTV, and in the range of −1.6 ± 3.4% and 1.1 ± 2.0% for all OARs (Fig. 4b).

### RT mask

**Fig. 5:**
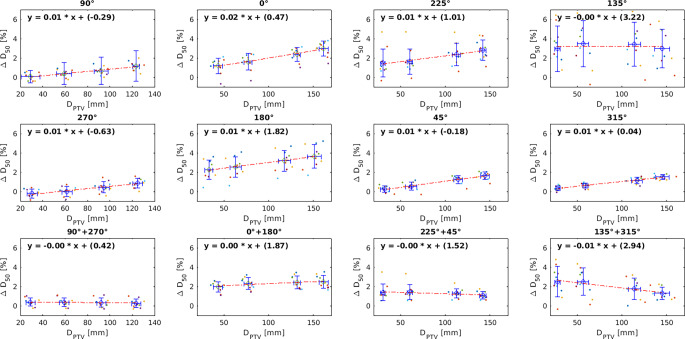
Percentage deviation ∆D_50_[%] in correlation to planning target volume (PTV) distance to the point of beam entrance on the skin or mask (*D*_*PTV*_) for different beam angles for 10 patients. *Circles* indicate the values at the mean D_PTV_ and the mean ∆D_50_[%] for each PTV. Horizontal and vertical *error bars* show the standard deviation of D_PTV_ and ∆D_50_[%], respectively

Figure 5 shows the ∆D_50_[%] in correlation to the *D*_*PTV*_ for all eight beam angles. Beams coming from angles 90°, 270°, 45°, and 315° were attenuated only by the RT mask and showed that ∆D_50_[%] increased with *D*_*PTV*_ (gradient of linear fit *m* *=* 0.01%/mm)_._ Beams coming from 90° and 270° entered through the left and right part of the RT mask and yielded the smallest mean ∆D_50_[%], with a minimum of −0.2 ± 0.5% (*D*_*PTV*_ = 30.4 ± 4.6 mm) and a maximum of 1.2 ± 0.5% (*D*_*PTV*_ = 123.5 ± 4.1 mm). Beams coming from 45° and 135° were larger in field size (10 × 10 cm) and yielded a minimum mean ∆D_50_[%] of 0.2 ± 0.3% (*D*_*PTV*_ = 32.6 ± 5.2 mm) and 1.6 ± 0.4% (*D*_*PTV*_ = 144.5 ± 5.0 mm), respectively.

The beam coming from beam angle 0° entered through the anterior part of the mask (with the thicker part around the nose) and showed a larger increase in ∆D_50_[%] (m = 0.02). The minimum mean ∆D_50_[%] was 1.2 ± 0.8% (*D*_*PTV*_ *=* 43.6 ± 5.0 mm) and the maximum was 3.0 ± 0.8% (*D*_*PTV*_ *=* 161.4 ± 5.1 mm). Meanwhile, the mean ∆D_50_[%] of opposing beams SF1 (90° + 270°) showed no correlation with the *D*_*PTV*_*. *The mean ∆D_50_[%] for SF1 was 0.3 ± 0.4%.

### RT table

Beams coming from the beam angle of 180° were attenuated by the RT table and the posterior part of the mask. The minimum mean ∆D_50_[%] was 2.2 ± 1.0% (*D*_*PTV*_ *=* 33.4 ± 4.4 mm) and the maximum was 3.6 ± 1.2% (*D*_*PTV*_ *=* 151.4 ± 7.6 mm). The mean ∆D_50_[%] of opposing beams SF2 (0° + 180°) showed no correlation with the *D*_*PTV*_*. *The mean ∆D_50_[%] for SF2 was 2.3 ± 0.6%.

### Mask holder

Beams coming from beam angles 225° and 135° were attenuated by the RT mask holder, the RT table, and the RT mask. The minimum mean ∆D_50_[%] was 1.5 ± 1.4% (*D*_*PTV*_ *=* 30.4 ± 2.6 mm) and the maximum was 2.8 ± 1.1% (*D*_*PTV*_ *=* 141.9 ± 3.5 mm) for the beam angle 225°; the minimum mean ∆D_50_[%] was 2.9 ± 2.4% (*D*_*PTV*_ *=* 27.9 ± 3.0 mm) and the maximum was 3.5 ± 2.4% (*D*_*PTV*_ *=* 56.7 ± 6.3 mm). Even though both beams were attenuated by the same auxiliary structures, the difference between the mean ∆D_50_[%] values was up to 1.4%. The largest differences in the range of 3.9–7.3% were found for four patients (P03, P04, P05, P09), while for the rest of the patients the ∆D_50_[%] was < 3.1%. After additional repositioning of the AST based on the visible auxiliary devices in the CT, the range of ∆D_50_[%] showed an improvement in the range of 0.4% (P04) and 1.9% (P05). No correlation with the *D*_*PTV*_ was observed for the sum of opposing beams SF3 (225° + 45), with a mean ∆D_50_[%] of 1.3 ± 0.6%. Nonetheless, for the sum of opposing beams SF4 (135° + 315°), a slight decline of the mean ∆D_50_[%] was observed with increasing *D*_*PTV*_ (m = −0.01), where the mean ∆D_50_[%] of SF4 was 2.0 ± 1.2%.

## Discussion

In an MRI-only workflow, the dosimetric validity of the sCTs is not only influenced by the body of the patient but also by the immobilization system, i.e., mask, mask holder, and tabletop. The dosimetric influence and the robustness of treatment plans against variation of these auxiliary structures and beam orientations were considered in the sCT-based dose calculation investigated in this manuscript, after a methodology was presented on how to include them in sCT-based treatment planning. A vast improvement in the dose differences was found by adding a mask structure surrounding the head of *d* = 3 mm and ρ = 1.2 g/cm^3^. Nonetheless, the mask’s form varies for each dataset and cannot be modeled accurately on the sCT for an MRI-only workflow; thus, the RT mask structure was a simplified model of the real mask (Fig. 1d), excluding the fixations to the mask holder, the mask extension, and the extra molded material on the anterior part. This may impact the accuracy of the calculated DVH comparison. The mean values and variation were larger than the results reported in the previous study [8], which evaluated dosimetric differences between CT- and sCT-based plans without considering the auxiliary structures. This implies that considering the auxiliary devices, including the RT mask, leads to a larger dosimetric variance. This can be caused by several factors, such as differences in auxiliary structures’ characteristics in sCT and CT, differences in HU or used HU LUT, AST placement accuracy, and the intrafractional variability of patient positioning for intracranial RT using a frameless RT mask.

The static field test evaluation showed the range of uncertainties that is caused by attenuation of each auxiliary structure. The uncertainties originated from the RT mask, including the anterior part close to the nose due to its larger thickness, the RT table, and the uncertainty of AST placement which was observed at beam angles 225° and 135°, where the beams were attenuated by the RT table, the mask holder, and the RT mask. An increase in ∆D_50_[%] with *D*_*PTV*_ when observing a single beam entrance can be caused by different factors. On one hand, this increase can be caused by differences in the auxiliary structures on CT and sCT, on the other hand, by differences in HU or HU LUT. Maspero et al. [20] reported that an inaccuracy of 0.7% can exist when using an incorrect HU LUT for sCT, while other factors such as registration inaccuracy between sCT and CT for the evaluation, etc., were deemed negligible. In this study, the same HU LUT as that used for generation of the sCT was used, and, hence, this uncertainty was minimized. To minimize the differences caused by the anterior part of the RT mask, possible approaches would include ensuring consistent thickness of the thermoplastic material if this structure is to be integrated into the AST, or to omit this step entirely.

Positioning of the AST was based on the visible silicone markers in the T1-VIBE DIXON and should be done before the dose calculation. While these markers were easily detected with the right window width/level setting, positioning the marker ROIs of the AST into the correct position required several iterations. Since the AST is a voxel-based volume, with every translation or rotation of the AST, it is newly interpolated onto the voxels of the new position. Consecutive interpolations may cause small structures to disappear. In this study, we found that the larger structures were still correctly displayed. Nonetheless, the influence of interpolation on the accuracy of dose calculation is yet to be investigated. The AST can easily be implemented in the daily clinical workflow and offers a solution for integrating auxiliary devices into treatment planning. Different possibilities exist to improve the quality of AST, e.g., by developing an automatic script for marker detection and AST placement, which is currently ongoing. By automating AST placement, interobserver variations can be minimized and the accuracy likely improved, since the number of AST translations/rotations will be low and, consequently, interpolation errors will be reduced.

Both beams from angles 135° and 225° were attenuated by the same structures which include the mask holder; however, the difference between the mean ∆D_50_[%] values was large. Furthermore, the RT mask holder additionally has two toggles for mask fixations. Since the mask holder is made of carbon, the accuracy of AST positioning is critical for dose comparison between the CT-based plan and the plan recalculated in the sCT, since the positions of the toggles in the AST will also differ to those in the CT-based plan. Repositioning the AST for four patients with the largest ∆D_50_[%] based on the visible auxiliary devices in CT showed improvement to up to 1.9%, which indicates that the accuracy of AST positioning has a large impact on the dose differences when compared to CT. Not only that, the registration inaccuracy between sCT and CT for the evaluation can still exist in the rotational direction, as reported in [8], possibly due to a suboptimal immobilization system position which could lead to a median rotational difference of ~1.5°. Hence, the large differences can be a consequence of this combination. Nevertheless, it must be noted that the position of the auxiliary devices in the CT (relative to the patient) cannot be considered the gold standard. The RT table structure was modeled on the CT as a template for the clinical treatment planning procedure in the TPS because the RT table at the treatment machine is different from the CT table. Since the position of the RT table structure template in the CT-based treatment plan varies and has not yet been investigated, the dose difference coming from a static field with a beam angle of 180° is only an indicator to determine the robustness of the sCT-based plan with respect to the RT table.

Similarly, the uncertainty of the mask holder structure position in the sCT was found to be in the order of one slice thickness, which is 1.5 mm (the resolution of T1-VIBE DIXON). In addition, variation in the relative position between the mask and the mask holder exists—not only in the RT setup used in the MRI but also in the CT. The intrafractional variabilities of patient positioning for intracranial RT using a frameless RT mask have been investigated in several studies [21–23]. Barnes et al. [23] reported that the patient’s motion was limited to approximately 3 mm and 3° by the RT mask. Nonetheless, the variation cannot necessarily only come from patient movement in the RT mask but also from the stability of the immobilization itself.

Evaluation of the clinical plan in the sCT showed that the dosimetric differences were still under 2% for OARs and target volumes, even for the four patients with the largest ∆D_50_[%] at beam angles 225° and 135°. A dosimetric significance of 2% was used in other studies [24], which is acceptable especially in the case of stereotactic radiosurgery, since a higher geometric accuracy can be achieved in an MRI-only workflow [8]. This indicates that even though the AST position is a source of dose variance, the dose difference is still tolerated. The results yielded by the sum of opposite static fields showed that the error partly canceled out and the values were constant along the *D*_*PTV*_*. *The correlation was only observed in the single static field beam. Since the clinical plans consisted only of VMAT and static arc plans and no static field plans, this indicates that using VMAT or static arc techniques with approximately full rotation should reduce the uncertainty coming from the auxiliary structures and thus increase the robustness of the sCT-based plan.

Statistical tests were not conducted due to the small sample size (10 patients). Hence, the results comparison in this study may be limited to this chosen patient set only and the found differences may be statistically insignificant. To reliably determine the statistical significance, the test should be conducted with a larger sample size. In comparison to this study, several other sCT studies did not acquire MRI in the RT setup and relied on the standard head coil and other immobilization techniques instead [16, 17, 25], because the studies focused more on the performance of the sCT algorithm rather than its clinical use. Other brain/head and neck sCT studies acquired MRI in the treatment position but did not consider the auxiliary devices in their evaluation [12–15]. An exception is reported by Lerner et al. [10], who evaluated another commercial deep learning-based sCT solution in combination with a different mask for the brain using a zero echo time sequence to determine the relative vertical position of the RT table for treatment planning. This technique was not adaptable in our case, due to the differing RT table characteristics at the MRI, CT, and treatment machine. We instead used embedded silicone markers to determine the relative position of the immobilization system and RT table. Dosimetrically, Lerner et al. [10] considered the attenuation caused by the RT mask to be small and hence negligible in the dose calculation. Controversially, in our study, we found that the RT mask has a dosimetric influence in the order of 1.5% and thus needs to be considered in dose calculation. This dosimetric influence depends on the RT mask material and needs to be specified for sCT-based treatment planning. In a similar study, Knäusl et al. reported the influence of the absence of an RT mask on a sCT-based carbon ion treatment [26], where a peak displacement of 0.2 cm was reported. To the best of our knowledge, to date, the effect of each auxiliary device has not yet been extensively investigated for MR-only photon beam RT.

Lastly, it is important to note that even though the variation in AST position can lead to dosimetric variance, the AST is not considered for daily positioning of the patient at the treatment machine. In the previous study, it was found that the sCT-based digitally reconstructed radiographs (DRR) were equivalent to the CT-based DRR [8], since the registration used in the kilovolt imaging system was based on bone structure only and thus the auxiliary structures were not considered. Hence, the variance of the auxiliary structures does not impact the accuracy of the sCT-based daily positioning using kilovolt imaging.

## Conclusion

This study proposed a technical solution for clinical implementation of a commercially available deep learning-based sCT by defining the immobilization system and RT table in the sCT as an auxiliary structure template. The auxiliary structures had a considerable dosimetric impact on the sCT-based RT and need to be considered during clinical treatment planning. The template can easily be implemented in the daily clinical workflow and offers a solution for integrating auxiliary devices in sCT-based treatment planning. Additionally, the dosimetric impact of auxiliary devices on sCT-based MR-only photon beam RT for brain was evaluated.
